# Decoupling Office Energy Efficiency From Employees' Well-Being and Performance: A Systematic Review

**DOI:** 10.3389/fpsyg.2019.00293

**Published:** 2019-02-20

**Authors:** Malgorzata W. Kozusznik, Laurentiu Paul Maricutoiu, José M. Peiró, Delia Mihaela Vîrgǎ, Aida Soriano, Carolina Mateo-Cecilia

**Affiliations:** ^1^Research Group for Work, Organizational and Personnel Psychology (WOPP), Katholieke Universiteit Leuven, Leuven, Belgium; ^2^Department of Psychology, West University of Timisoara, Timisoara, Romania; ^3^Research Institute IDOCAL, University of Valencia, Valencia, Spain; ^4^Instituto Valenciano de Investigaciones Económicas, Valencia, Spain; ^5^Instituto Valenciano de Edificación, Valencia, Spain

**Keywords:** energy-efficiency, well-being, performance, office buildings, systematic review, decoupling

## Abstract

Energy efficiency (i.e., the ratio of output of performance to input of energy) in office buildings can reduce energy costs and CO_2_ emissions, but there are barriers to widespread adoption of energy efficient solutions in offices because they are often perceived as a potential threat to perceived comfort, well-being, and performance of office users. However, the links between offices' energy efficiency and users' performance and well-being through their moderators are neither necessary nor empirically confirmed. The purpose of this study is to carry out a systematic review to identify the existing empirical evidence regarding the relationships between energy-efficient solutions in sustainable office buildings and the *perceptions* of employees' productivity and well-being. Additionally, we aim to identify relevant boundary conditions for these relationships to occur. A systematic literature search of online databases for energy efficiency literature (e.g., *Environment Complete, GreenFILE*), employee literature (e.g., *PsycINFO, Business Source Complete*) and general social science literature (e.g., *Academic Search Complete*) yielded 34 empirical studies. Also, inclusion and exclusion criteria were set. The results suggest that it is possible to decouple energy costs from organizational outcomes such as employee well-being and performance. Also, they indicate the existence of moderators and mediators in the relationship between green office building solutions and well-being/performance. Directions for future research and the implications for practice considering different stakeholders interested in implementing green building solutions, adopting energy-saving measures in offices, and improving employees' functioning are suggested.

## Introduction

Maintaining the current pace of energy consumption in office buildings to ensure users' comfort can have catastrophic outcomes for natural systems and society (Marchal et al., 2011). Based on our emissions of carbon dioxide (CO_2_), one of the major greenhouse gases responsible for global warming, and other greenhouse gases into the atmosphere, current projections estimate that there will be an increase in the Earth's temperature of between 1.0 and 3.7°C during the twenty-first century (Anderson et al., 2016). It is not sustainable to maintain the levels of comfort and performance in offices while using energy the way we do now.

Energy efficiency has become a perennial issue for contemporary organizations. It can be understood as the ratio of performance output (e.g., thermal comfort) to energy input (Erbach, 2015), or in other words, “getting the most out of every unit of energy you buy” (Herring, 2006, p. 11). From an environmental perspective, improving energy efficiency would lead to reducing CO_2_ emissions (Worrell et al., 2001), lowering office buildings' carbon footprint and, therefore, counteracting climate change. Among the heaviest consumers of energy are office buildings, ranging from 100 to 1,000 kWh/m^2^ per year in Europe (Dubois and Blomsterberg, 2011). In Spain, a yearly average of 170 Kwh/m^2^ is consumed only for heating, cooling, and lighting (Eurostat, 2002). According to the Third Report on the State of the Energy Union (EC, 2017), the services sector (which includes, among others, offices, wholesale/retail, healthcare, education, and accommodation/food) accounted for 13.6% of the total energy consumption in the EU-28 countries in 2015. In Spain, office buildings are the second leading source of energy consumption within this sector, with a consumption equal to 31.7% in 2014 (Ministerio de Fomento, 2017). The increased energy use in offices in recent years may be attributed to factors such as the growth in information technology, air conditioning, or density of use (Schneider Electric, 2006).

Since the mid-2000s, energy has become an EU priority, and the focus on energy efficiency in different countries has recently increased, following EU initiatives (Nilsson, 2012) that seek cost-efficient ways to make the European economy more climate-friendly. Specifically, as part of its long-term energy strategy, the European Commission set targets for 2020, 2030, and 2050, aiming at energy savings of 20, 32.5, and 80–95%, respectively, compared to the 1990 levels (2012/27/EU Directive, 2012^1^; EC, 2018a,b). Moreover, the Paris global agreement (UNFCCC, 2015), signed in 2015 and aimed at avoiding the effects of climate change (Rogelj et al., 2016) through sustainable solutions, increased worldwide sensitivity to the issue of energy.

On top of climate mitigation effects (Levine et al., 2007), implementing energy-efficient solutions can have clear benefits for companies (van Doren et al., 2016) in terms of lowering costs. Indeed, energy is an important operational expenditure for office buildings. In the U.S., it represents about 19% of total overhead for the typical office building (U.S. National Grid, 2002), and in Spain it equals 135.000€ per year spent on energy in an average office building with 5.000 m2 (Asociación de Empresas de Eficiencia Energética, 2013). The benefits of introducing energy-efficient solutions can be great, and they can help offices save between 45 and 55% of their energy costs (Cuchí and Sweatman, 2013).

Despite these undeniable benefits, sustainable energy conservation measures to reduce energy consumption in office buildings are not adopted widely enough in organizations (Lovins, 1992; van Doren et al., 2016), due to some existing barriers. From the point of view of the employer, it is still not clear whether energy efficient solutions in office buildings are beneficial for office users in terms of satisfaction and performance. In this case, an important barrier is limited awareness and information about financial, health, well-being, and comfort benefits of energy-efficient solutions (Levine et al., 2007; UNEP, 2009; Boardman, 2010; Immendoerfer et al., 2014; van Doren et al., 2016). Managers or owners of office buildings are often reluctant to implement energy efficient solutions, due to their concerns about keeping employees productive in a comfortable environment (Schneider Electric, 2006) with adequate indoor environmental quality (IEQ) conditions (e.g., acoustics, lighting, air quality; Astolfi and Pellerey, 2008; Wong et al., 2008; Lai and Yik, 2009; Frontczak and Wargocki, 2011), which involves using electrical equipment. This resonates with an old misconception that energy efficiency requires sacrifices in terms of users' comfort (Komor and Katzev, 1988; OTA, 1992). In general, all of this suggests that it has not been made clear enough that companies can save money because of using energy-efficient solutions in offices while ensuring their employees' well-being and performance. Therefore, it is a challenge to make them see the possibility of *decoupling* high levels of comfort and performance from high energy-related costs.

### Theoretical Underpinnings for Decoupling Energy Costs From Well-Being and Performance in Office Users

Some theoretical considerations explain the circumstances under which it is possible to decouple high levels of comfort and performance from high energy-related costs. The theory of person-environment fit (Kaplan, 1983) focuses on the human-environment interface, the supportive role of environments for basic processes (e.g., perception, attention), and attainment of goals. It is based on the analysis of environments as a source of necessary action (Barker, 1968) and the concept of “behavior-environment congruence” (Wicker, 1973), which considers supportive workplaces to be essential for people's functioning, rather than a source of pressure and constraint. Accordingly, based on person-environment fit theoretical considerations, an office environment that is adequate for the tasks carried out at work can improve user comfort and foster performance (Vischer, 2007). This is consistent with the work characteristics approach (Hackman and Oldham, 1976), according to which the design of optimally challenging work characteristics has to take the person into account in order to motivate employees internally and increase their performance.

It is important, however, when implementing decoupling with energy-efficient solutions, to remember to take into account the concept of environmental fit or misfit (see Alexander, 1970; Zeisel, 2005), which conceptualizes environmental misfit as inappropriate or excessive demands placed on users despite their adaptation and adjustment behaviors (Vischer, 2007). Thus, environmental factors in the office (e.g., lighting and daylighting, noise and noise control, office furniture, and office spatial layout) should not place additional demands or overload the office users.

Based on these theories, decoupling energy costs from well-being and performance is possible, as long as the fit between the office environment and the office user is ensured. This is, however, not an easy task. Human factors may play an important role in meeting these challenges by complementing the design process (Thatcher, 2012) of sustainability-oriented, “ergonomically and ecologically optimized” (Steimle and Zink, 2006 p. 2358) solutions, and still being able to produce high satisfaction and performance.

### Employees' Well-Being and Performance in Sustainable Office Buildings

Employee well-being and performance are broad categories that include workplace affect (e.g., positive or negative affect, comfort), attitudes (e.g., job satisfaction), motivational variables (e.g., work engagement, work self-efficacy), and cognitive factors that are relevant to both performance and health. The category of employee health is also broad, and it includes both subjective (e.g., psychological well-being) and objective (e.g., health complaints) health. Similarly, employee performance encompasses different facets, such as task performance, contextual performance, creative and adaptive performance, and counter-productive behavior [e.g., absenteeism; see Koopmans et al. (2011) for a systematic review]. The evaluation of these performance facets may be subjective (either self-assessed or appraised by relevant others such as the supervisor, team members, or clients) or objective (e.g., number of errors on proofing tasks).

The growing requirement of environmental comfort to enhance employees' performance in office buildings is often accompanied by an increase in energy consumption. However, sustainability needs pose the challenge of managing energy consumption in a way that should lead to energy savings, a task that is especially urgent (Lu et al., 2016). The improvement of energy efficiency would lead to reducing CO_2_ emissions from fuel and electricity uses (Worrell et al., 2001) and, therefore, to counteracting climate change. Specifically, political and scientific challenges include the reduction of CO_2_ emissions by lowering consumption and using clean energies. Unlike what is generally believed, reducing energy costs should not necessarily mean compromising good environmental quality. In fact, the aim of sustainable offices is to be *both* energy-efficient and ensure good environmental quality. Indeed, the essence of energy efficiency is “using less energy to provide the same or improved level of service to the energy consumer in an economically efficient way” (Goldman et al., 2010). Nowadays, increasing energy efficiency in buildings has become one of the main strategies to achieve reduced energy consumption (Nagy et al., 2015).

Sustainability of office buildings is a multi-faceted concept that involves economic sustainability (e.g., maintenance and energy costs), environmental sustainability (e.g., energy demand and energy consumption), and employee-related sustainability (e.g., turnover, job performance, job satisfaction, well-being). When dealing with sustainability issues in office buildings, researchers use terms such as “high quality buildings” (Roulet et al., 2006), “energy-efficient buildings” (Amasyali and El Gohary, 2016), “high performance buildings” (Day and Gunderson, 2015), “sustainable buildings” (Keyvanfar et al., 2014; Bluyseen et al., 2016), “sustainable design” (Steemers and Manchanda, 2010), or “green buildings” (Newsham et al., 2013; Menadue et al., 2014). These terms are often juxtaposed or (often inaccurately) interchanged. What they all have in common is that they refer to buildings that *aim* to be energy-efficient while maintaining the health (and often the performance) of their users. In the present review, we refer to all of these terms as “sustainable buildings.”

In practice, do sustainable buildings manage to reach their aim? In other words, is it possible to maintain office workers' well-being and productivity while ensuring the energy efficiency of office buildings and, therefore, decouple energy costs from well-being and productivity? In this case, decoupling refers to ways for businesses to improve their outcomes (e.g., employees' performance and well-being) while shrinking their ecological footprints (e.g., being energy efficient), and it has been suggested as the central challenge of our age (KPMG, 2012).

### The Impact of Energy-Efficient Solutions on Employees' Well-Being and Performance

Until now, the relationship between office energy efficiency and employees' outcomes (e.g., well-being and performance) has been far from clear. On the one hand, stimulating the energy efficiency of buildings is advocated in order to enhance public expenditure savings across Europe and improve occupant well-being (IEA, 2014), and some research shows that energy-saving or sustainable interventions in office buildings can improve employee well-being and performance (Agha-Hossein et al., 2013). There is empirical evidence suggesting that energy-efficient solutions in office buildings have clear potential to ensure person-environment fit. These solutions often include features aimed at creating a flexible work environment with appropriate spaces for different tasks, such as open plan offices, meeting rooms, areas for concentration (Agha-Hossein et al., 2013), which increase the fit between the person and the environment. For example, good-quality lighting supports interpersonal communication and visual performance, which are essential for office users' comfort (Nagy et al., 2015) and performance. Additionally, there are a number of existing practical building solutions stemming from advances in sustainable technologies that ensure better IEQ conditions by improving air quality (Fisk, 2000), lighting, acoustics, privacy, and personal workspace (Leder et al., 2016; Ornetzeder et al., 2016), while reducing density (Fisk, 2000) and improving the workplace image and esthetics (Newsham et al., 2013). All these features can have an impact on the fit between the office space and the office user.

On the other hand, there is also research that shows that employees working in sustainable buildings have lower levels of well-being than employees in traditional buildings (Menadue et al., 2014). This may be due to the fact that some energy-efficient solutions designed to reduce costs (e.g., occupancy sensors, automatic blinds, or central HVAC systems) drastically limit the office user's control over his/her working environment (Kozusznik et al., 2017), and they generate environmental and behavioral conditions that jeopardize well-being (Boyce et al., 2006; Rashid and Zimring, 2008). This lack of control, according to the occupational stress theory of demand-control (Karasek, 1979), limits an important job resource that may help employees to deal with demands (Vischer, 2007), and which, following the principles of reactance theory (Brehm, 1966), can negatively impact well-being and performance (McCoy and Evans, 2005).

Finally, some research studies reported no significant relationship between green buildings and changes in office users' well-being and performance (Thatcher and Milner, 2014). These mixed results indicate that it is still unclear whether sustainable office buildings may ensure both energy efficiency and the well-being and performance of their users.

In this review, we focus on organizational outcomes (i.e., job performance and well-being) for three reasons. First, from a theoretical point of view, the analysis of effective and efficient decoupling requires the consideration of the most important outputs in the environment of work and organizations. Work performance is of great interest for organizations and for the individual, especially when both performance and well-being are promoted. The previously described theoretical models consider performance and well-being to be highly related and dependent on the person's environment (Alexander, 1970; Hackman and Oldham, 1976; Kaplan, 1983; Zeisel, 2005), and they explain the theory behind the connection between the antecedents (energy efficiency) and these outcomes. Second, the pragmatic reason is that most of the research in the area of decoupling applied to work environments focuses on both outcomes. Finally, “ad hominem,” if we can obtain evidence about the feasibility of decoupling with good results in terms of well-being and performance, it could be an important motivator for employees and organizations to engage in actions that would lead to decreased energy expenditure, promote environmental sustainability, and, at the same time, maintain adequate levels of well-being and performance.

## The Present Review

The purpose of the present work is to carry out a systematic review to identify and synthesize the existing empirical evidence on the relationships between energy-efficient solutions in sustainable office buildings and employees' well-being and productivity (performance and absenteeism, especially sick-leave). Additionally, we aim to identify relevant boundary conditions for these relationships to occur. In PICOS terms, we are interested in finding out whether the introduction of energy efficient technologies (i.e., intervention) increases the levels of well-being and job performance (i.e., outcomes) of employees who work in office spaces (i.e., population), compared to pre-intervention levels (of well-being and job performance), or compared to employees from buildings without energy-efficient technologies.

In order to address these objectives, we analyzed the literature on employee reactions (in terms of well-being and performance) to sustainable buildings. We also analyzed literature on employee reactions to specific energy-efficient solutions often adopted in sustainable buildings to reduce lighting and thermal energy costs.

The present review expands the knowledge on the outcomes of innovative and sustainable building systems and technologies because it adopts a user-centered approach by considering the well-being and performance of office users, which in turn can bring economic benefits to the organization. Therefore, the added value in this review is that it focuses on research studies that used data provided by the participants (e.g., the perception of comfort), rather than research studies that used simulations or mathematical models without collecting any data from the occupants. This review can also provide additional and valuable evidence about decoupling energy consumption and costs from well-being and performance. Showing existing data about decoupling well-being and performance from energy-related costs would make it possible to provide the market with evidence so that office builders and managers can be more confident about introducing energy efficient solutions in office buildings to reduce costs and improve important outcomes for employees. It may also show the boundary conditions under which these solutions may be more effective.

## Literature Search and Study Selection

The purpose of this review is to investigate the empirical evidence regarding the employees' appraisal and behaviors in terms of well-being and performance when cost- and energy-efficient technologies are used in office spaces.

To achieve this goal, we selected relevant online databases for energy efficiency literature (e.g., *Environment Complete, GreenFILE*), employee literature (e.g., *PsycINFO, Business Source Complete*) and general social science literature (e.g., *Academic Search Complete*). The full list of these databases is presented in Appendix 1, and we conducted the systematic search in September 2016. The following databases provided the most numerous results: *PsycINFO, Scopus, MEDLINE, Academic Search Complete, Academic Search Premier, Vocational Studies Premier, Environment Complete, CINAHL Plus, Business Source Complete, GreenFILE, Professional Development Collection, and Art and Architecture Complete*.

The search query included keywords relevant to cost-efficient technologies (“decoupling,” “energy saving,” “energy consumption,” “energy conservation,” “energy efficien^*^,” “low energy,” “low-energy”), and a wide range of possible employee reactions (“well-being,” “stress,” “physical complaints,” “job satisfaction,” “emotion^*^,” “mood,” “affect,” “comfort,” “absenteeism,” “presenteeism,” “turnover,” “retention,” “productivity,” “performance,” “engagement,” “counterproductive”). We restricted the initial results to the target population, using keywords such as “employee,” “office,” “personnel,” “staff,” “white collar.” Finally, we included keywords relevant for selecting empirical research papers (“research,” “results,” “participants,” “subjects,” “correlation^*^,” “statistic^*^”). In the search engine, we used the option “all fields” for all keywords, except for the “office” keyword, which was restricted to manuscript abstracts. We did not use any additional restrictions regarding the study design, year of publication, country of publication, or population demographics. The entire search phrase is presented in the Annex 1.

Two independent evaluators analyzed 377 abstracts resulting from the online search, using the following eligibility criteria: (a) the abstract had to mention that a research study is reported in the paper; (b) the abstract had to mention measurements of workplace characteristics and measures of psychological variables. In addition to these two inclusion criteria, we also used an exclusion criterion: we discarded all abstracts that mentioned occupations that were not office-based (e.g., medical personnel, teachers, astronauts, construction workers), and we excluded any paper that did not assess *perception* of comfort. The two evaluators had an agreement rate of 90.45% (i.e., they both agreed to reject 310 abstracts, and both agreed to accept 31 abstracts, and they made divergent decisions in the case of 36 abstracts). For the next stage, we retained all the articles selected by at least one evaluator; therefore, we searched for the full-text version of 67 manuscripts.

Following the analysis of the abstracts, we searched for the full-text versions of the titles selected, and we found 61 manuscripts. In this stage, we retained research papers that: (a) collected behavioral or self-reported data from employees; (b) addressed the energy consumption issue (i.e., the employees worked in at least one energy-efficient setting); and (c) reported quantitative data. However, 33 of the full-text papers were not included in the review because researchers did not report any data from human participants (i.e., self-report measures or job performance measures), or they did not address the energy consumption issue. The online search was supplemented by a manual search of the papers published by the *Energy and Buildings* journal between 1977 and 2016, which yielded 6 additional eligible papers.

**Figure 1:**
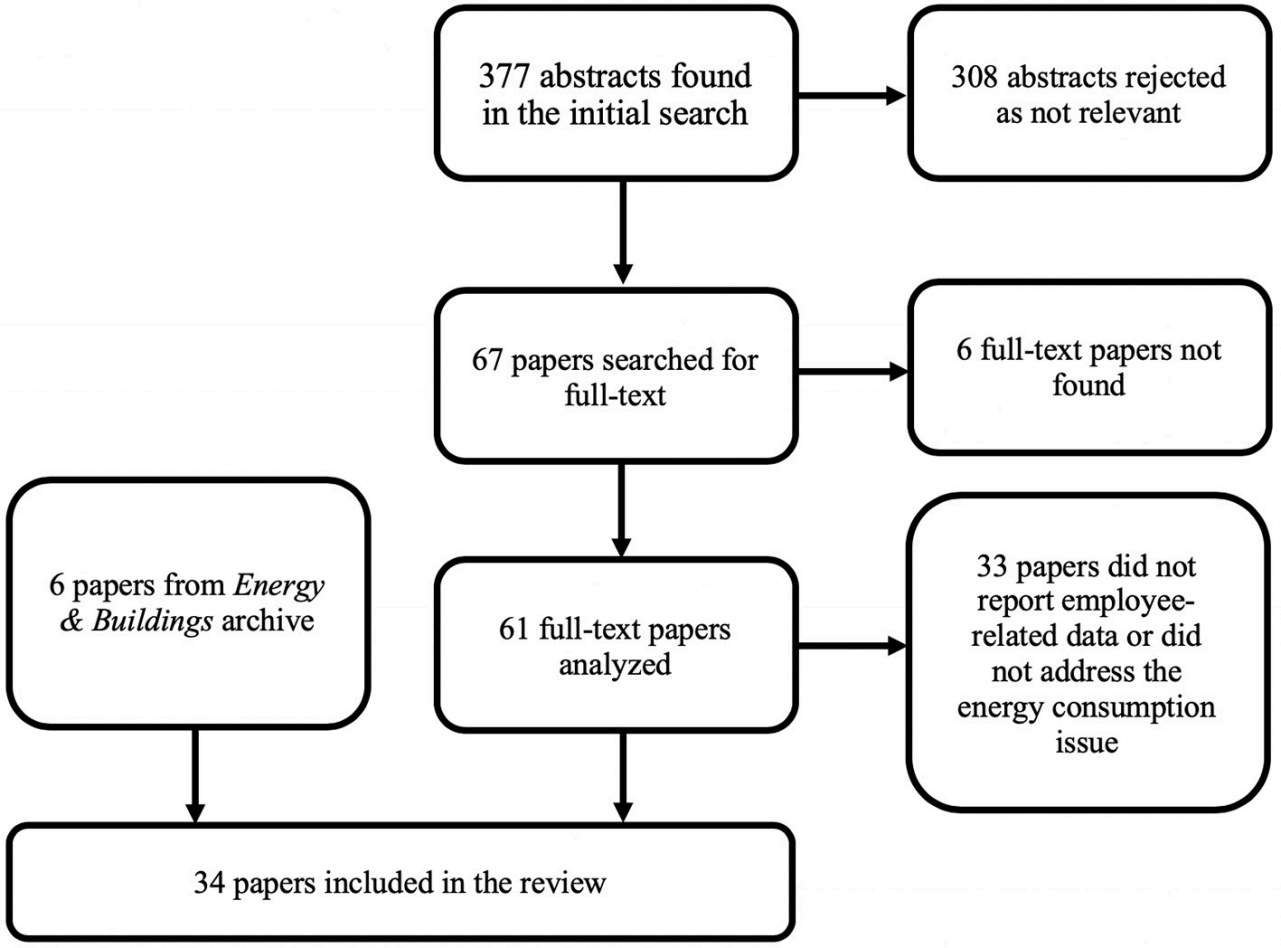
Flowchart of the papers in the review.

Therefore, 34 research papers are included in this review, and a general representation of the entire process of analysis and selection of the research papers is shown in Figure 1.

In the next section, we will present the results reported in the papers identified in the literature search. Based on their main focus, these results can be classified in the following three groups: (a) employees' reactions (in terms of well-being and performance) to sustainable offices; (b) employees' reactions to specific energy-efficient solutions aimed at reducing lighting costs; and (c) employees' reactions to specific energy-efficient solutions aimed at reducing thermal costs. These specific areas of sustainable solutions are related to some of the most important indoor environmental quality (IEQ) conditions, namely, thermal, visual, and air quality (Wong et al., 2008; Lai and Yik, 2009; Frontczak and Wargocki, 2011), which are often the main dimensions considered in IEQ research to study office environments (e.g., Veitch et al., 2007).

## Results

### Employee Reactions to Sustainable Offices

Energy-saving, sustainable interventions in buildings involve the introduction of new, energy-efficient technologies into existing office buildings. The studies identified in the systematic literature search that focus on employees' reactions (in terms of well-being and performance) to sustainable offices adopted three types of designs. First, there are longitudinal studies that follow office users' reactions over time in green and conventional office buildings and compare them. Second, there are comparative cross-sectional studies that focus on the users of both types of office buildings. Finally, there are descriptive studies that include the sample of green buildings only to describe their users in terms of employee well-being and performance. The research also indicates some boundary conditions in the relationship between sustainable buildings and well-being. Please see Table 1 for an overview of the studies included in this section.

**Table 1:** Employee Reactions to Sustainable Offices.

**References**	**Study design**	**Measure of well-being**	**Measure of performance**	**Definition of Green buildings**	**Main results**	**Moderators/mediators**
Agha-Hossein et al., 2013	Longitudinal design, pre- (*n =* 162) and post- (*n =* 183) occupancy surveys in one old and one refurbished buildings, both located in London, UK.	Work environment satisfaction, and the perception of well-being and enjoyment at work as being positively affected by the work environment	Perception of productivity as positively affected by the work environment.	Refurbished office building with flexible work environment (e.g., open plan offices, meeting rooms, areas for concentration) and energy-efficient features (e.g., the lighting system incorporating sensors, centrally controlled services).	In the refurbished building, the results show decreased carbon emissions and increased job satisfaction, self-reported productivity, and well-being.	Reducing the control of the office users over their environment (that helped the company to save energy) did not have a significant negative impact on users' performance.
Thatcher and Milner, 2014	One-year longitudinal comparison of two groups of office employees: group 1 moved into a green building, group 2 stayed in a conventional building. Measurements were taken in both groups shortly before (T1), 6 months after (T2) and 1 year after (T3) the move. Respondent from group 1: 98 (T1), 80 (T2), and 59 (T3); respondents from group 2: 114 (T1), 41 (T2), and 52 (T3), all from South Africa.	Physical and psychological well-being, job satisfaction	Perceived productivity	The green building was one of the first GreenStar-accredited buildings in South Africa.	There was no increase in perceived productivity nor physical and psychological well-being among the employees who moved to the new buildings in comparison to employees who remained in the conventional buildings.	
Thatcher and Milner, 2012	Longitudinal comparison of two groups; 1) a group that moved into South Africa's first GreenStar-accredited building (161 respondents) and 2) a group that did not (79 respondents). Measures were taken twice: before the move (Time 1) and 6 months later (Time 2).	Physical and psychological wellbeing, job satisfaction	Perceived productivity	The green building was the first GreenStar-accredited buildings in South Africa.	There were no significant differences between Time 1 and Time 2 on measures of perceived productivity, psychological wellbeing, physical wellbeing or job satisfaction in either group.	
Murtagh et al., 2013	Pre- and post-intervention surveys, energy measurement and disposal of feedback for 18 weeks post-baseline, and two focus groups in 83 university office workers.	Positive attitudes toward reducing energy use at work		Provision of feedback by an application (MyEcoFootprint) regarding each participant's energy use by showing red, amber or green light depending on the user′s energy efficiency in the previous week. Possibility to see detailed statistics (e.g., for hour) and historical data up to 10 weeks, to compare against the average usage for their office, and to receive hints on saving energy.	Attitudes to energy conservation were negatively related to energy use.	Individual feedback can improve their energy behavior and engage in energy reduction actions.
Liang et al., 2014	Longitudinal, post-occupancy study with surveys administered every month during 7 months that compared 3 green (134 respondents) and 2 conventional (99 respondents) office buildings in middle Taiwan.	Environmental satisfaction (i.e., satisfaction with the quality of the acoustics, lighting, thermal comfort, indoor air, and overall IEQ in the building).		Green buildings were accredited by the green building certification system (EEWH) in Taiwan	There were significantly higher levels of satisfaction in green buildings in comparison to conventional buildings (the difference was only quantitative, not qualitative). Employees in both types of buildings provided positive evaluations of environmental satisfaction, thus, the difference is not in terms of valence (e.g. neutral vs. positive evaluations).	The office users sharing a concern on energy conservation were more amenable to slightly deficient IEQ, which means that they perceived greater thermal comfort than the group of lesser concern.
Newsham et al., 2013	Comparative, post-occupancy online survey among 2545 occupants of 12 green and 12 conventional office buildings, in Canada and northern United States.	Environmental satisfaction, visual and physical discomfort symptoms, mood, and sleep quality	Organizational commitment, turnover intent	Buildings that obtained LEED (Silver, Gold, Platinum), Go Green Plus or various certificates.	In green buildings, there was higher overall environmental satisfaction with ventilation and temperature, aesthetic appearance, size of workspace, and access to a view of outside. Also, green office users reported lower frequency of visual and physical discomfort symptoms, as well as better mood, and better sleep quality at night. There were no significant differences between building types (green vs. conventional) in organizational commitment, turnover intent, or job satisfaction.	
Menadue et al., 2014	A comparative, post-occupancy internal environment monitoring and occupant survey evaluation in 4 ‘green-rated’ and 4 ‘non-green-rated’ commercial office buildings in Adelaide, South Australia.	Visual, thermal, and aural comfort.	Productivity	Green buildings were possessing 4 or 5 Green Star rating certification.	Green-rated buildings exhibit equal or decreased occupant satisfaction of internal thermal conditions when compared to non-green-rated buildings.	
Leder et al., 2016	A comparative study of occupant satisfaction in 12 green and 12 conventional buildings in Canada and northern United States	Occupant satisfaction with IEQ, job satisfaction		Buildings holding LEED certification, having applied for it, holding an alternative certification, or whose owner have taken substantial and documented steps toward sustainability.	Being in a green building was associated with: (a) greater satisfaction with IEQ (i.e., acoustics and privacy, lighting, ventilation and temperature), after accounting for other workstation factors (i.e., its size, having an office with full-height walls and a door); and (b) overall environmental satisfaction. Building type (green vs. conventional) was not related to occupants' job satisfaction.	
Roulet et al., 2006	A comparative study in 64 office buildings divided into two groups: “low energy” buildings and “high energy” buildings in nine European countries.	Perceived comfort, perceived health		“Low energy” buildings with an energy performance index lower than the median	On the average, the occupants perceive low energy buildings as significantly more comfortable and healthier than the high energy ones.	Perceived control over the environment is positively and significantly correlated with the perceived comfort.
Amasyali and El Gohary, 2016	A comparative online panel survey of 618 residential and office building occupants in Arizona (AZ), Illinois (IL), and Pennsylvania (PA), United States.	Thermal comfort, visual comfort, IAQ, health	Personal productivity	Energy Star and LEED certified buildings.	Occupants of Energy Star certified buildings were more satisfied only with environmental protection and energy cost saving than occupants of non-Energy Star buildings. There were no significant differences in the levels of satisfaction in the users of LEED and non-LEED certified buildings.	Office building users who had control over the environment (e.g., adjusted thermostats, used/adjusted room air conditioning units, and used/adjusted ceiling fans) were more satisfied with thermal comfort in summer. Users of private workspaces reported higher satisfaction with thermal comfort (in winter and in summer) and visual comfort.
Mokhtar Azizi et al., 2015	A comparative study between 2 green buildings and 2 conventional buildings occupants (total of 270 respondents), New Zealand.	Occupants complaints to the building manager		Green buildings with the design intent to be energy efficient (one held certification from GreenStar New Zealand), including such energy efficient features as highly glazed windows, layered facades, or occupancy sensors.	Green building occupants complain less to the building manager in comparison to their counterparts from conventional buildings.	
Lin et al., 2016	A comparative study in 10 green (360 respondents) and in 8 traditional (244 respondents) office buildings in China	Satisfaction with IEQ		Green buildings with a three-star green building label certification	Green building occupants reported higher satisfaction with the overall indoor environment quality, thermal environment, indoor air quality, and office lighting that the traditional building. There were no differences in the levels of satisfaction with the acoustic environment in the two types of buildings.	Satisfaction with building service performance is significantly higher in green office building than in traditional office building.
Steemers and Manchanda, 2010	Monitoring and surveys of 12 office buildings in the UK and India (correlational study).	Occupant satisfaction, comfort, frequency of ailments (e.g., dry eyes, sore throat, headache), overall perception of health		Sustainable building design consisted of the degree of mechanization defined in terms of two basic categories: air conditioned and naturally ventilated.	There was no direct relationship between the energy use (or CO2 emissions) of buildings and occupant satisfaction.	The degree of control of the environment was evaluated as one of the most important aspects in determining office users' satisfaction.
Tsushima et al., 2015	Survey questionnaires in 7 electricity-saving office buildings in the Kanto region, Japan	Satisfaction with the IEQ	Productivity	Electricity-saving office buildings were characterized by a 15% reduction in peak power consumption	The implementation of electricity saving in a proper way by the office users did not decrease their satisfaction.	Workers who were aware and supportive of saving electricity, reported significantly greater satisfaction with IEQ and performance.
Ornetzeder et al., 2016	Quantitative (*n =* 231 respondents) and qualitative field study (20 interviews, 1 focus group, site visit protocols and photo material) in two low-energy office buildings in Austria.	Satisfaction with the room temperature, air quality, lighting, noise and personal workspace		Green buildings were designed according to low-energy standards and certified according to one of the two national standards (klimaaktiv or TQB).	The occupants of green buildings were satisfied with the room temperature, air quality, lighting and noise conditions, and with the personal workspace.	The overall employees' satisfaction is related with the evaluations of the services provided by the facility management. Female users were significantly less satisfied with the perceived temperature conditions than male users. Employees in leadership positions were significantly more satisfied with all comfort-related parameters than persons not holding a leadership position.
Ng and Akasah, 2013	Post occupancy survey evaluation in 3 energy-efficient buildings (111 respondents) in Malaysia.	Occupants' comfort		Green buildings included different types of buildings made to reduce energy consumption and cope with the problems derived from the over consumption of natural resources (mostly coal).	The majority of occupants were not satisfied with the thermal comfort and lighting conditions of the buildings, even if these buildings were certified with sustainable building rating tools.	
Lawrence and Keime, 2016	Post occupancy evaluation in two green buildings (101 respondents), Sheffield, UK.	Comfort		Buildings using the SchneiderElectric Sigma System as a Building Management System (BMS).		Employees' self-reported comfort can be improved by providing occupants with the control over their environment.
Day and Gunderson, 2015	Survey data from 8 high performance buildings (118 respondents) in the United States			High-performance buildings included those that used various sustainable strategies to reduce overall energy use, optimize all installed systems, and to promote health and productivity for its occupants		Employees who reported having received effective training for high-performance building features were significantly more likely to be satisfied with their office environment as compared to those who did not receive it.
Keyvanfar et al., 2014	Systematic literature review, expert input study.			Sustainable buildings included those with a variety of features for sustainability assessment (e.g., energy efficiency, water management, waste management, land use), covering the greenery/environmental issues, with consideration on economic and social-friendly approaches.		The authors show the role of users' adaptive behavior in energy efficient buildings to increase their satisfaction. They provide 18 adaptive behaviors significant for the cooling system in energy efficient indoor environments, and different 18 adaptive behaviors important for the lighting system.

#### Longitudinal Studies

Regarding the longitudinal studies, all of them showed that sustainable office buildings have neutral to positive effects on users' well-being and performance. Agha-Hossein et al. (2013) assessed the evolution of energy consumption and employee's job satisfaction, well-being, and perceived productivity, following the refurbishment of an office building in London. The refurbishment aimed at creating a flexible work environment with appropriate spaces for different tasks (e.g., open plan offices, meeting rooms, areas for concentration). Some energy-efficient features included incorporating sensors in the lighting system, as well as centrally controlled services that kept employees from having control over their immediate environment. The results of a *t*-test analysis showed that 6 months after starting to use the refurbished building, there were significant reductions in carbon emissions and significant improvements in job satisfaction, self-reported productivity, and well-being (Agha-Hossein et al., 2013).

Furthermore, Murtagh et al. (2013) investigated the effect of individual feedback on energy use at the work desk, and they also tested the relationships between individual determinants, energy use, and energy reduction in 83 office workers at a university. The research design comprised pre- and post-intervention surveys, energy measurement, feedback for 18 weeks post-baseline, and two focus groups. Results of correlation analyses emphasized that no individual variables were related to energy reduction, and only attitudes toward energy conservation were related to energy use, so that the more positive the attitudes, the less energy used. The authors concluded that people used much more energy than they needed at their work desks, and that individual feedback improved their energy behavior and engagement in energy reduction actions.

Similarly, Thatcher and Milner (2014) conducted a longitudinal study using data collected shortly before, 6 months after, and 1 year after the move from conventional buildings (more than 30 years old, complying with local building legislation) to new green buildings (one of the first GreenStar-accredited buildings in South Africa). They found no increase in perceived productivity or physical and psychological well-being among the employees who moved to the new buildings. In a similar study, Thatcher and Milner (2012) investigated a group of 161 employees who moved into South Africa's first GreenStar-accredited building and a group of 79 employees who remained in a conventional building. The measurements were taken twice: before the move (Time 1) and 6 months later (Time 2). The authors found no significant differences between Time 1 and Time 2 on measures of perceived productivity, psychological well-being, physical well-being, job satisfaction, or absenteeism in either group. In both studies, the analyses employed did not explore the time-group interaction. Therefore, the results should be viewed with caution.

Finally, Liang et al. (2014), in a post-occupancy study with surveys administered every month for 7 months, found significantly higher levels of satisfaction in green buildings accredited by the green building certification system in Taiwan (known as EEWH), compared to conventional buildings, but the difference was only quantitative, not qualitative. Employees in both types of buildings provided positive evaluations of environmental satisfaction, and so the difference was not in the valence (e.g., neutral vs. positive evaluations).

#### Transversal Comparative Studies

Comparative studies that adopted a cross-sectional design show more diversified results than studies with a longitudinal design. On the one hand, there are studies that indicate a positive or neutral impact of sustainable buildings on office users' well-being. First, Newsham et al. (2013), in their online survey of occupants of 24 green and conventional office buildings across Canada and the northern United States, showed that occupants of green buildings reported higher overall environmental satisfaction, especially related to: ventilation and temperature, aesthetic appearance, size of workspace, and access to a view of outside. In addition, occupants of green buildings reported lower frequency of visual and physical discomfort symptoms, as well as better mood and better sleep quality at night. There were no significant differences between building types (green vs. conventional) in organizational commitment, turnover intent, or job satisfaction. However, occupants of green buildings indicated that their facilities offered a better workplace image.

In a recent study, Leder et al. (2016) attempted to identify relevant parameters for occupant satisfaction and explore the effects of office type (open-plan vs. private) and building type (green vs. conventional). Buildings were classified as green if they held LEED certification, had applied for it, held an alternative certification, or the owner had taken substantial and documented steps toward sustainability. The results showed that, after accounting for other workstation factors (i.e., its size, having an office with full-height walls and a door), being in a green building was associated with greater satisfaction with specific environmental characteristics such as acoustics and privacy, lighting, ventilation, and temperature. It was also associated with overall environmental satisfaction (Leder et al., 2016). However, building type (green vs. conventional) was not related to occupants' job satisfaction (Leder et al., 2016).

Roulet et al. (2006) divided a sample of 64 office buildings into two groups: “low energy” buildings (with an energy performance index below the median) and “high energy” buildings (with an energy performance index above the median) to determine whether these buildings differed on perceived comfort, perceived health, and energy use. The results showed that, on average, the occupants perceived low energy buildings as significantly more comfortable and healthier than high energy ones. These results suggest that it is possible to design and build office buildings that are simultaneously energy efficient, healthy, and comfortable.

Amasyali and El Gohary (2016) used online surveys of 618 residential and office building occupants to investigate occupants' satisfaction levels with the values related to energy use behavior and energy consumption in residential and office buildings (i.e., thermal comfort, visual comfort, IAQ, health, personal productivity, environmental protection, and energy cost saving). The authors compared these satisfaction levels in Energy Star and non- Energy Star certified buildings, as well as in LEED and non-LEED certified buildings. The results showed that occupants of Energy Star certified buildings, both residential, and office buildings, were more satisfied with environmental protection and energy cost saving than occupants of non-Energy Star buildings. However, they found no significant differences in the satisfaction levels of occupants of LEED and non-LEED certified buildings.

Lin et al. (2016) conducted a satisfaction survey in green and traditional office buildings and concluded that green building occupants reported higher satisfaction with the overall indoor environment quality, the thermal environment, the indoor air quality, and the office lighting. Interestingly, there were no differences in the levels of satisfaction with the acoustic environment in the two types of buildings.

Finally, in their comparative study, Mokhtar Azizi et al. (2015) revealed that green building occupants complained less to the building manager compared to their counterparts from conventional buildings. These authors also indicated an advantage of working in an energy-efficient building that consists of increasing occupants' awareness of the environmental impact of their thermal adjustment (i.e., turning on the cooling or the heat system).

On the other hand, there are also cross-sectional comparative studies that show negative effects of green buildings on their users' well-being and performance. For example, Menadue et al. (2014) conducted a post-occupancy evaluation of some “green-rated” and “non-green-rated” commercial office buildings in Adelaide, South Australia. The research design combined internal environment monitoring and occupant surveys to assess the perceived and actual visual, thermal, and aural comforts, as well as health and productivity. The results showed that green-rated buildings exhibited equal or decreased occupant satisfaction with internal thermal conditions compared to non-green-rated buildings. The authors suggest that green-rated buildings should be designed and operated to meet the long-term comfort of the occupants, along with the immediate need for certification credits and the long-term efficiency of the building systems.

In a similar vein, Steemers and Manchanda (2010) demonstrated the relationships between sustainable building design (depending on the degree of mechanization defined in terms of two basic categories: air conditioned and naturally ventilated) and occupant well-being, based on monitoring and surveys of 12 office buildings in the UK and India. The authors collected total energy consumption characteristics of the buildings and converted them to CO_2_ emissions per year by applying conversion factors of 0.19 for gas, 0.25 for oil, and 0.43 for electricity (DEFRA, 2003). They tested whether energy use and CO_2_ emissions were correlated with occupant satisfaction and comfort. The results demonstrated that increased energy use in the case study buildings was associated with reduced occupant control, and this was related to reduced occupant comfort and satisfaction. Moreover, the reported health of occupants (i.e., ailments, their frequency, and the overall perception of health) correlated with their levels of satisfaction. Although the authors did not test the mediation chain between these variables, their suggestion was that more energy does not improve well-being.

#### Descriptive Studies

The third group of studies on the effects of green buildings on office users in terms of well-being and performance includes descriptive studies that focused only on green buildings. These studies indicate mixed results regarding green office users' comfort and productivity.

On the one hand, Tsushima et al. (2015) administered survey questionnaires to investigate the comfort and productivity of occupants in seven electricity-saving office buildings (based on a 15% reduction in peak power consumption) in the summers of 2011–2013, after the Great East Japan Earthquake of 2011. The results showed that workers learned to implement electricity saving in a proper way that did not decrease their comfort. For example, excessive indoor air temperatures (like 28°C) were avoided, and the desk level illuminance substantially decreased (from 750 lux to around 400 lux). The authors suggest that the key factor to improve workers' comfort and productivity is raising their awareness, and that building devices that induce the occupants' energy-saving actions are vital for energy saving. Ornetzeder et al. (2016) reported the results of a qualitative and quantitative field study in two office buildings in Austria, designed according to low-energy standards. The results showed that the occupants were satisfied with the room temperature, air quality, lighting and noise conditions, and with the personal workspace. Although no comparison was made with a traditional building, this finding supports the idea that low levels of energy use in office buildings can be aligned with high levels of well-being.

On the other hand, Ng and Akasah (2013) conducted a survey of energy-efficient buildings in Malaysia to identify the problems affecting occupants' comfort and buildings' IEQ. The results showed that the majority of the occupants were not satisfied with the thermal comfort and lighting condition of the buildings, even if these buildings were certified with sustainable building rating tools.

#### Boundary Conditions

The literature review showed that the degree of occupants' control moderated the relationship between sustainable offices and the occupants' outcomes in several studies. For example, Lawrence and Keime (2016) showed that employees' self-reported comfort can be improved by providing occupants with control over their environment. Similarly, Amasyali and El Gohary (2016) showed that office building occupants who adjusted thermostats, used/adjusted room air conditioning units, and used/adjusted ceiling fans were more satisfied with thermal comfort in summer. In turn, regarding performance, Agha-Hossein et al. (2013) did not find an association between control over the environment and employees' performance. Specifically, they showed that disempowering employees (or reducing their control over their environment) helped the company to save energy without having a significant negative impact on employees' performance.

There are other moderators identified in the literature in the relationship between sustainable offices and employees' outcomes. First, Day and Gunderson (2015) investigated existing occupants' training in high-performance buildings to provide recommendations for future occupants' education. They indicated that employees who reported having received effective training in high-performance building features were significantly more likely to be satisfied with their office environment compared to those who did not receive it. Second, Keyvanfar et al. (2014) studied “user satisfaction from adaptive behavior,” which is an aspect of user satisfaction that refers to restoring comfort when a change in the situation hampers it. In their two-step study, they provided 18 adaptive behaviors that were significant for the cooling system in energy-efficient indoor environments, and 18 other adaptive behaviors that were important for the lighting system. This list of user satisfaction adaptive behaviors draws our attention to the role of users' adaptive behavior in energy-efficient buildings to increase their satisfaction. Third, Amasyali and El Gohary (2016) showed that users of private workspaces reported higher satisfaction with thermal comfort (in winter and in summer), visual comfort, and environmental protection. Finally, Ornetzeder et al. (2016) showed that overall employees' satisfaction was related to the evaluations of the services provided by the facility management.

#### Summary of Employees' Reactions to Sustainable Offices

We can see from the results mentioned above that the five longitudinal studies report three significant positive relationships and two non-significant relationships between green buildings and their users' reactions. However, the results from the cross-sectional comparative studies are more varied. Specifically, they show that, when comparing the two types of buildings at only one time point, the same number of non-significant and significant positive relationships is found between green buildings and users' reactions (5 positive, 5 non-significant), as well as one negative relationship. The most diverse results stem from descriptive studies that only used a sample of green buildings. In this case, out of three studies, one positive, one neutral, and one negative relationship between green buildings and office users' responses were found. These diverse results for different types of designs suggest that study design is an important factor that may have an impact on the results obtained. Longitudinal designs are considered superior to cross-sectional or descriptive designs for several reasons. Specifically, they offer pre- and post-occupancy measurements, which makes it more reasonable to consider the unidirectionality of causal effects, and they reduce the risk of common-method variance when using self-report measures (Podsakoff et al., 2003). It is important to emphasize that the longitudinal studies identified in this review did not use more robust analyses that are more appropriate for these designs, such as repeated-measures ANOVA or Longitudinal Growth Curve Modeling, which can model change.

In summary, although we can identify research that shows that green buildings can have detrimental outcomes for office occupants in terms of well-being (Steemers and Manchanda, 2010; Ng and Akasah, 2013; Menadue et al., 2014), the majority of the studies included in this review suggest that green office buildings improve employees' well-being (Roulet et al., 2006; Leder et al., 2016; Ornetzeder et al., 2016) and performance (Agha-Hossein et al., 2013). There is also research that indicates that green buildings have a neutral effect on well-being (Lin et al., 2016). Finally, the research points out some moderators in the relationship between sustainable buildings and employees' outcomes, such as: the degree of occupants' control over their office environment (Steemers and Manchanda, 2010; Agha-Hossein et al., 2013; Amasyali and El Gohary, 2016; Lawrence and Keime, 2016), users' adaptive behavior in energy-efficient buildings (Keyvanfar et al., 2014), services provided by the facility management (Ornetzeder et al., 2016), effective training in high-performance building features (Day and Gunderson, 2015), or the use of private workspaces (Amasyali and El Gohary, 2016).

### Employee Reactions to Solutions Aimed at Reducing Lighting Costs

Electric lighting is one of the major costs for any building. Lighting is estimated to represent about 19% of the total generated electricity (IEA, 2006), and it accounts for 30 to 40% of the total energy consumption in office buildings (Halonen et al., 2010). The research on sustainable solutions in the domain of lighting has predominantly focused on looking at the impact of introducing blinds systems (e.g., blinds, roller shades), the use of lighting control systems in offices through light sensors, and implementing more efficient lighting scenarios that involve reductions in the lighting power density to produce more illuminances with less energy use. The main challenge of this research domain is to find the optimal balance between allowing daylight into offices (to reduce electric lighting costs) and providing enough shade (to avoid the rising costs of cooling the building). Table 2 summarizes the reviewed papers on the impact of sustainable lighting solutions on office users' outcomes.

**Table 2:** Employee reactions to solutions aimed at reducing lighting costs.

**References**	**Study design**	**Measure of well-being**	**Measure of performance**	**Definition of sustainable solutions**	**Main results**	**Moderators/mediators**
Kang et al., 2013	Comparative study on 15 subjects in Korea in two scenarios: the one in which temperature was controlled by the integrated control and the control one.	Thermal and lighting comfort		A system that integrated air-conditioning system, lighting system and blind system	There were energy savings of 19.6% for lighting in a system that integrated air-conditioning system, lighting system and blind system. In the same time, building occupants declared thermal and lighting comfort.	
Meerbeek et al., 2014	Comparative study of automated vs. manual blinds system in an office building in Netherlands	Satisfaction with the overall indoor climate		Automated or manual blinds system	No significant differences in the comfort ratings between automated mode and manual mode users, both user groups being reasonably satisfied with the overall indoor climate	The most frequent reason for manual override the automatic mode of the blinds system was glare discomfort, low daylight entrance, or the need to create the outside view. The most adjustments are made in the morning.
Konis, 2013	Introduction of roller shades to an office building in San Francisco, US.	Satisfaction with the building, satisfaction with the workspace		The introduction of daylight to reduce electrical lighting energy consumption and to enhance Indoor Environmental Quality and to minimize the need for mechanical cooling and lighting.	Introduction of roller shades to an office building in San Francisco led to a 12.6% decrease in lighting costs, while employees remained generally satisfied with their workspace and the building overall.	-Orientation (perimeter, NW, core zone): Employees in the perimeter zone reported sufficient visual comfort, while the majority of participants in the NW perimeter zone showed low levels of visual comfort. The majority of employees working in core zones of the building perceived insufficient levels of daylight. -Grade of shading: windows that were shaded more than 50% generated dissatisfaction. The highest satisfaction levels were recorded when roller shades covered up to 30% of the window.
Nagy et al., 2015	Comparative study of three different control modes of lighting	Lack of complaints from the employees as an indirect measure of satisfaction with lighting.		An energy-efficient strategy for controlling lighting in office spaces that involve the use of infrared motion and light sensors to make decisions regarding the use of electric lights, or regarding the intensity of electric lights.	The authors reported 37.9% energy savings and interpreted the lack of complaints from the employees as an indirect measure of satisfaction with lighting.	
Linhart and Scartezzini, 2011	Comparative study on 20 subjects in two energy-efficient lighting scenarios.	Subjective visual comfort	Visual performance	Two energy-efficient scenarios, scenario 1 with LPD of 3.9 W/m2 (more efficient) scenario 2 with LPD of 4.5 W/m2.	No significant differences in the computer-based performance tasks under the two lighting scenarios where found. Performance in the paper-based performance task was significantly better under the scenario 1 than under the scenario 2. The subjective evaluation of both scenarios was generally neutral-to-positive	

#### Studies on Sustainable Blind Systems

The first and most numerous group of studies includes research on office users' reactions to blinds systems. In their study, Kang et al. (2013) described an integrated blind, lighting, and air-conditioning system, and they reported energy savings of 40.8% for cooling and 19.6% for lighting. At the same time, building occupants declared thermal and lighting comfort. Similarly, Konis (2013) reported that the introduction of roller shades in an office building in San Francisco led to a 12.6% decrease in lighting costs, while employees remained generally satisfied with their workspace and the building overall. Finally, Meerbeek et al. (2014) analyzed how employees were using the automated blinds system in an office building in Netherlands, and they found that 73.6% of blinds adjustments were user adjustments, and only 26.4% of blinds adjustments were commanded by the automated system. The results showed no significant differences in the comfort ratings between automated mode and manual mode users, with both user groups being reasonably satisfied with the overall indoor climate.

#### Studies on the Use of Sensors

The second type of sustainable lighting solution in office buildings includes lighting controlled by light sensors. In this domain, Nagy et al. (2015) described an energy-efficient strategy developed to control lighting in office spaces, which consisted of combined information from infrared motion sensors (to determine whether the room was occupied or not) and light sensors that turned the electric lights on or off depending on the illuminance level provided by daylight. The system combined this information to make decisions about the use of electric lights or their intensity. The authors reported 37.9% energy savings and interpreted the lack of complaints from the employees as an indirect measure of satisfaction with lighting.

#### Studies on Implementing More Efficient Lighting Scenarios

Finally, reducing the lighting power density is a simple strategy for diminishing lighting costs, but it is unclear how much one can reduce this environmental parameter without negatively affecting the employees' behaviors and well-being. To clarify this, Linhart and Scartezzini (2011) compared the visual performance and subjective visual comfort of 20 subjects in two lighting scenarios: scenario 1 with LPD of 3.9 W/m^2^ (more sustainable) and scenario 2 with LPD of 4.5 W/m^2^. The authors reported that no significant differences were found on the computer-based performance tasks in the two lighting scenarios. However, performance on the paper-based performance task was significantly better in scenario 1 than in scenario 2. The subjective evaluation of both scenarios was generally neutral-to-positive. These results suggest that it is possible to reduce lighting costs without jeopardizing users' visual comfort and performance.

#### Boundary Conditions

Some studies pointed out moderators in the relationship between sustainable lighting solutions and occupants' outcomes, such as glare, the location of the employee in the building, and the degree of shading. Indeed, the most frequent reason for manual override of the automatic mode of the blinds system was glare discomfort, low entrance of daylight, or the need for an outside view (Meerbeek et al., 2014). The results also showed that most adjustments are made in the morning, and correlations between outside weather and blind adjustments are too weak to permit accurate predictions (Meerbeek et al., 2014). Moreover, Konis (2013) observed that there were differences in satisfaction with some facets among the employees working in different office zones. For example, the employees in the perimeter zone reported that they perceived sufficient levels of daylight to work comfortably with the electrical lighting turned off. This was not the case for the majority of the participants in the NW perimeter zone, who showed low levels of overall satisfaction with visual comfort. In addition, the majority of employees working in core zones of the building perceived insufficient levels of daylight. Finally, the author reported that between 55 and 73% of the employees shade their workplace. The results also suggest that there is a minimum necessary degree of shading (50%) to produce office users' satisfaction, whereas the highest satisfaction levels were recorded when roller shades covered up to 30% of the window.

#### Summary of the Employee Reactions to Sustainable Lighting Solutions

In sum, the evidence collected suggests that the solutions aimed at reducing lighting costs are effective in reducing these costs, while maintaining (Kang et al., 2013; Konis, 2013; Meerbeek et al., 2014; Nagy et al., 2015) or improving (Linhart and Scartezzini, 2011) office users' comfort and performance. This was demonstrated in the studies that focused on three different types of sustainable solutions, that is, introducing blinds systems, sensors, and efficient lighting sources that produce more illuminance with less energy. This evidence is relevant for the decoupling issue because it indicates that it is possible to maintain office users' well-being and productivity while ensuring the energy efficiency of office buildings and, therefore, decoupling energy costs from well-being and productivity. In this way, using energy-efficient lighting solutions is a way for businesses to improve their outcomes (e.g., employees' performance and well-being) while shrinking their ecological footprints (e.g., using less energy for lighting).

### Employee Reactions to Solutions Aimed at Reducing Thermal Costs

Strategies aimed at reducing thermal costs involve careful planning and design of new heating or cooling facilities, reductions in thermal losses by providing improved thermal insulation, or modifying indoor temperature. In the literature on sustainable solutions in the domain of temperature, we can distinguish two main types of studies. On the one hand, there are studies that focus on the effects of implementing local devices (e.g., chairs, desks, ceiling panels) aimed at improving thermal comfort. On the other hand, there is research that focuses on global thermal solutions in office buildings, which include ventilation, cooling, and air conditioning solutions. The latter category points out some boundary conditions in the relationship between sustainable solutions and well-being and performance. Table 3 includes a summary of the reviewed papers on the impact of sustainable thermal solutions on office users' outcomes.

**Table 3:** Employee reactions to solutions aimed at reducing thermal costs.

**References**	**Study design**	**Measure of well-being**	**Measure of performance**	**Definition of sustainable solutions**	**Main results**	**Moderators/mediators**
Pasut et al., 2013	Evaluation of the subjective responses of 30 subjects to a heated/cooled individual chair carried out eight times (before, during, and after a break period, at 20-min intervals) in four test conditions (chamber temperatures set at 16, 18, 25 and 29°C).	Thermal sensation, comfort, temperature satisfaction		Heated/cooled individual chair	The results showed that the heated/cooled chair provided thermal comfort under all tested conditions, both warm and cool, strongly influencing the subjects' thermal sensation and comfort.	
Pasut et al., 2015	Comparison of subjective responses of 23 college students to the heated/cooled chairs placed in a chamber in three conditions: temperatures of 16°C, 18°C, and 29°C.	Subjective responses for thermal sensation and comfort		Heated/cooled individual chair	The heated/cooled chair improved thermal comfort, and perceived air quality. The chair provided comfortable conditions for 92% of the subjects in a range of temperatures from 18°C to 29°C.	
Zeiler et al., 2014	“Human-in-the-loop” design. Measurements taken in two room temperatures (22°C and 19.5°C) in 20 employees during six weeks on an office floor.	Skin temperature of the office occupants measured by an infrared (IR) device		IR devices located above office desks that measured the skin temperature of occupants' fingers. If the IR devices identified discomfort, additional personalized heating of 98W per occupant was provided using a radiation panel to improve thermal sensation.	The “human-in-the-loop” control strategy generated more than 20% energy savings on heating demand, and up to 40% energy savings on cooling demand, without cool discomfort being consciously felt by the occupant.	
Heise and Huafen, 2013	A field survey study on 214 participants in an office building in the USA.	Occupant thermal comfort		Office space heated and conditioned with a radiant ceiling panel (RCP) system	The installed RCP system was able to provide thermal comfort to occupants of the office building.	During the cooling season, in the same temperature, females reported greater cold sensation than males and therefore felt less comfortable.In the same temperature: (1) people between 30 and 50 years old feel more thermal comfort than older or younger workers; (2) people working far from a window feel more thermal comfort.
Fisk et al., 2012	Comparison of four office scenarios with modified outdoor air ventilation rates.	Health	Annual economic benefits, work performance, short-term absence.	Office scenarios with increased minimum ventilation rates (VRs).	Increasing minimum ventilation rates (VRs) improved annual economic benefits, health, work performance, and decreased short-term absence.	
Indraganti et al., 2013	Thermal comfort field study in four office buildings in Tokyo for 3 months in summer 2012. 435 occupants who returned 2402 questionnaires	Thermal comfort		Following the ‘setsuden’ (energy saving) campaigns, which promoted the minimum indoor temperature setting of 28°C in summer.	Thermal acceptability was 76% in naturally ventilated (NV) mode and 92% in air-condition (AC) mode. In AC mode 84% subjects voted comfortable on the sensation scale.	
Valancius and Jurelionis, 2012	The analysis of air temperature swings and the calculation of energy consumption in four offices (oriented either to North–East or to South–West).	Subjective thermal evaluations	Employees' productivity	A refurbished real office building with natural ventilation installed.	Optimal office temperature to decrease energy costs and maximize long-term performance should be set to 21.6°C, should be gradually decreased to 18°C one hour before the end of the work hours, and should be set to this temperature throughout the night.	
Pfafferott et al., 2007	Analysis of German office buildings in summer and winter climate	Occupants' comfort		Passively cooled low-energy office buildings	Buildings which use only natural heat sinks for cooling provide good thermal comfort during typical and warm summer periods. However, long heat waves overstrain passively cooled buildings with air-driven cooling concepts in terms of thermal comfort.	Passively cooled low-energy office buildings can provide acceptable thermal comfort in German summer climate, as long as the outside temperature is not extreme. Occupants reported less satisfaction with the room temperature in summer (as compared with the ratings they provided in winter).
Wagner et al., 2007	A survey of 50 occupants of 16 German low energy office buildings during typical summer periods.	Thermal comfort and occupant satisfaction with indoor environments		Low energy office buildings that were naturally ventilated or passively cooled	During typical summer periods in Germany, about 75% of the office occupants rated the indoor climate as neutral or better.	Both in summer and winter the occupants' perceived that the control of the indoor climate has a strong influence on the satisfaction with thermal indoor conditions.
Kang et al., 2013	Comparative study on 15 subjects in Korea in two scenarios: the one in which temperature was controlled by the integrated control and the control one.	Thermal and lighting comfort		A system that integrated air-conditioning system, lighting system and blind system.	The results show energy savings of 40.8% for cooling in a system that integrated air-conditioning system, lighting system and blind system. In the same time, building occupants declared thermal and lighting comfort.	
Kuchen and Fisch, 2009	A survey for winter periods, in 148 workspaces belonging to 25 office buildings in Germany.	Thermal comfort		Defining the optimal temperature that can be attained by assessing the relationship between the measured data and the subjective data given by the users.	The users of office buildings expressed acceptance to pre-established thermal conditions.	

#### Local Thermal Solutions in Office Buildings

In the domain of local thermal solutions in office buildings, Pasut et al. (2013) evaluated a heated/cooled individual chair for its effect on thermal sensation and comfort in a chamber with four different temperatures. Subjective responses about thermal sensation, comfort, and temperature satisfaction were obtained eight times before, during, and after a break period, at 20-min intervals. The results showed that the heated/cooled chair provided thermal comfort under all tested conditions, both warm and cool, strongly influencing the subjects' thermal sensation and comfort. In a similar study, Pasut et al. (2015) tested the subjective responses about the thermal sensation and comfort given by college students, who tested the heated/cooled chairs placed in a chamber, with different temperatures: 16°C, 18°C, and 29°C. The results showed that the heated/cooled chair positively influenced the subjects' thermal sensation and improved their thermal comfort and perceived air quality. The chair provided comfortable conditions for 92% of the subjects in a range of temperatures from 18°C to 29°C.

Most of the research studies on air temperature combine information from temperature sensors, estimations of clothing insulation, and subjective ratings of thermal comfort. Instead of relying on temperature sensors and subjective measures, Zeiler et al. (2014) used an infrared (IR) device to measure the skin temperature of the office occupants. This information, collected in real-time, was used to optimize the temperature control system, by introducing the “human-in-the-loop” (Zeiler et al., 2014). In their original approach to thermal comfort research carried out in winter, they placed IR devices above office desks and measured the skin temperature of occupants' fingers. Then, the authors decreased the overall room temperature from 22°C to 19.5°C. If the IR devices identified discomfort, additional personalized heating of 98 W per occupant was provided using a radiation panel to improve the local- (hands) and overall thermal sensation. Zeiler et al. (2014) reported that the “human-in-the-loop” control strategy generated more than 20% energy savings in heating demand, and up to 40% energy savings in cooling demand, without discomfort being consciously felt by the occupant. These results are consistent with Heise and Huafen (2013), who conducted a field study on occupant thermal comfort in an office space that was heated and conditioned with a radiant ceiling panel (RCP) system in an office building in the USA. The results of a thermal comfort survey administered to regular occupants of this building showed that the installed RCP system was able to provide satisfying thermal comfort to occupants in this building.

#### Global Thermal Solutions in Office Buildings

There are also several studies on global thermal solutions implemented in office buildings. In this domain, Fisk et al. (2012) tested four office scenarios with modified outdoor air ventilation rates, which is a relevant strategy for increasing energy efficiency. They showed that increasing minimum ventilation rates (VRs) improved annual economic benefits, health, and work performance, and decreased short-term absence. The results suggest that it is possible to attain positive organizational and employee outcomes in conditions of decreased building energy consumption.

In addition, Valancius and Jurelionis (2012) investigated the impact of temperature variation, a simple strategy to reduce costs in energy consumption, on employees' productivity in a refurbished real office building with natural ventilation installed. Using the subjective thermal evaluations provided by the employees, Valancius and Jurelionis (2012) concluded that the optimal office temperature to decrease energy costs and maximize long-term performance should be set to 21.6°C, gradually decreased to 18°C 1 hour before the end of the work day, and left at this temperature throughout the night. These results show that decreasing energy costs while maintaining performance levels is feasible.

Pfafferott et al. (2007) investigated occupants' comfort in passively cooled low-energy office buildings, and they concluded that these buildings can provide acceptable thermal comfort in a German summer climate, as long as the outside temperature is not extreme. Interestingly, Pfafferott et al. (2007) concluded that occupants reported less satisfaction with the room temperature in summer (compared to the ratings they provided in winter), although the thermal sensation was similar. Based on this finding, the authors (Pfafferott et al., 2007) suggested that long-term measurements of thermal comfort are necessary.

In agreement with these results, Kuchen and Fisch (2009) conducted a survey on thermal comfort in winter periods, in 148 workspaces belonging to 25 office buildings, in order to define an optimal operative temperature. The authors combined the objective measures for temperature and humidity with the subjective data obtained by questionnaires about thermal aspects of the close environment within the office. The results showed that the users of office buildings expressed acceptance of pre-established thermal conditions, even though the thermal prediction index (as proposed by the Norm—Predicted Mean Vote—which functions in Germany), indicated that these thermal conditions were not comfortable.

In turn, Indraganti et al. (2013) conducted a thermal comfort field survey in 28 office buildings in India that followed the “setsuden” (energy saving) advice of the government. The authors showed that thermal acceptability was 76% in naturally ventilated (NV) mode and 92% in air-condition (AC) mode. In AC mode, 84% subjects voted comfortable on the sensation scale. The authors also showed that, regarding the energy savings, using air conditioning only during temperature excursions proved to be an efficient method for reducing power consumption.

Finally, Wagner et al. (2007) surveyed the occupants of 16 German low energy (i.e., naturally ventilated or passively cooled) office buildings, and showed that, during typical summer periods in Germany, about 75% of all office occupants rated the indoor climate as neutral or better.

#### Boundary Conditions

The studies on global sustainable thermal solutions in offices indicate some boundary conditions in the relationship between sustainable solutions and well-being and performance. First, the research suggests again that, regardless of the time of the year, perceived control is an important moderator between the objective measures and subjective ratings of indoor temperature. Indeed, Wagner et al. (2007) showed that the occupants' perceived control over the indoor climate has a strong influence on their satisfaction with thermal indoor conditions.

Second, climate may be a determinant factor for the moderating role of sex, age (Heise and Huafen, 2013; Indraganti et al., 2013), or weight (Indraganti et al., 2013). On the one hand, in a warm humid wet climate in summer, in the same thermal conditions, women, young subjects, and thin people perceived greater thermal comfort than men, older people, and obese occupants, respectively (Indraganti et al., 2013). On the other hand, in the same temperature during the cooling season, females reported a greater sensation of cold and therefore less comfort than men (Heise and Huafen, 2013). Moreover, in the same temperature, people between 30 and 50 years old felt more thermal comfort than older or younger workers (Heise and Huafen, 2013). Other research that highlights the importance of climate suggests that office buildings with sustainable thermal solutions can maintain thermal comfort in summer, as long as the outside temperature is not extreme (e.g., during heat waves in the summer; Pfafferott et al., 2007).

Finally, outside window proximity can also be a determinant factor for thermal comfort (Heise and Huafen, 2013). Specifically, people working far from a window perceived more thermal comfort than those working close to a window (Heise and Huafen, 2013).

#### Summary of Employee Reactions to Sustainable Thermal Solutions

In general, all the research in the group of studies on local solutions shows that locally implemented devices are able to ensure the thermal comfort of office users in a wide range of temperatures. In turn, the group of studies on global solutions that, in addition to comfort, also focus on work performance, show more varied results. In general, these studies indicate that the impact of these solutions on well-being and performance is moderate to positive. The mechanism behind the positive association between local solutions and office users' well-being may be that local solutions can be seen as an additional individual resource to deal with the environment that gives them a sense of greater control over their environment.

In sum, sustainable office solutions aimed at reducing thermal costs in offices can maintain well-being (Pfafferott et al., 2007; Heise and Huafen, 2013; Zeiler et al., 2014) or increase it (Pasut et al., 2013), and also increase the performance of employees (Fisk et al., 2012), while, at the same time, reducing thermal costs. Future research in this area should take into account the role of perceived control, climate and/or season, age, sex, weight, and location in the office as potential moderators in the relationship between sustainable thermal solutions in offices and the outcomes of office users.

## Discussion

The purpose of the present study was to carry out a systematic review of the existing research to in order identify and synthesize the existing empirical evidence on the relationships between energy-efficient solutions in sustainable office buildings and employees' productivity and well-being. Additionally, we aimed to identify relevant boundary conditions for these relationships to occur. To achieve this goal, we conducted a systematic search of the online databases that yielded 387 articles, of which 34 articles were included in this review.

As we can see, the majority of the studies reviewed (31) suggest neutral (e.g., Pfafferott et al., 2007; Heise and Huafen, 2013; Kang et al., 2013; Konis, 2013; Meerbeek et al., 2014; Zeiler et al., 2014; Nagy et al., 2015; Lin et al., 2016) to positive (Roulet et al., 2006; Linhart and Scartezzini, 2011; Fisk et al., 2012; Agha-Hossein et al., 2013; Pasut et al., 2013; Leder et al., 2016; Ornetzeder et al., 2016) effects of green building solutions on employees well-being and performance. However, in our systematic review, we identified three studies that indicate that green buildings have detrimental effects on office employees in terms of well-being and performance (Steemers and Manchanda, 2010; Ng and Akasah, 2013; Menadue et al., 2014).

The results indicate that green building solutions can generally be a good measure to diminish energy costs without jeopardizing well-being and performance. Furthermore, they suggest that decoupling could be slightly more effective in maintaining or increasing office users' well-being and performance in the case of sustainable solutions applied to thermal and lighting conditions, compared to general energy-saving sustainable interventions in office buildings. However, the results cannot be considered conclusive because we identified some design limitations in a number of studies. Thus, we will make several suggestions for future research that would make it possible to further clarify the relationship between green office buildings and users' outcomes.

First, the studies reviewed show the existence of moderators and suggest some mediators in the relationship between green office building solutions and well-being /performance. These moderators include the degree of occupants' control over their office environment, users' adaptive behavior, the perception of the services provided by the facility management, effective training in high-performance building features, the access to and use of private workspaces, and the location of the employee in the building.

The issue of occupants' control is an especially important avenue for future research, and it could be studied as both a moderator and a mediator in the relationship between sustainable office building solutions and employee outcomes. On the one hand, the occupational stress theory of demand-control (Karasek, 1979) may shed light on the moderating role of (lack of) control. According to this theory, control is an important job resource that may help employees to deal with demands (Vischer, 2007). The demands can be understood as environmental factors in the office (Vischer, 2007), such as lighting, temperature, or office spatial layout. These work characteristics can be optimally used to improve performance (Hackman and Oldham, 1976), or when inappropriate or excessive, they can overload office users (Vischer, 2007). Employees may benefit from many sources of control, and having the opportunity to manipulate the devices to control the temperature, noise, privacy, furniture location, etc., in their work environment may contribute to the efficient use of energy and, thus, improve performance and well-being. In contrast, following the principles of reactance theory (Brehm, 1966), the features of efficient solutions in office buildings that limit the users' control (e.g., occupancy sensors that control automatic lighting, automatic blinds systems, or central HVAC systems) can negatively impact well-being and performance (McCoy and Evans, 2005). Therefore, the subjective perception of environment control is important for employees because their reactions become more positive (Lee and Brand, 2005). In fact, office users who perceived that they had adequate control over their indoor climate reported greater comfort and less building-related symptoms (Boerstra and Beuker, 2011). Moreover, research by Leaman and Bordass (1999) shows that users perceive work performance to be higher in buildings where they have more control over their environments (e.g., temperature, ventilation). On the other hand, (lack of) control could explain some mediating relationships between sustainable office solutions and employee outcomes. Indeed, lack of control of the work environment is a demotivating factor with a negative impact on well-being and performance (McCoy and Evans, 2005). Learned helplessness provides a theoretical framework that helps to explain the connection between (lack of) perceived control over the environment and motivation (see Evans and Stecker, 2004, for a review). Specifically, it explains that, when attempts to cope with environmental stressors fail repeatedly, a person can develop a belief in non-contingency, induced by repeated exposure to this uncontrollable stressor, that will generalize beyond the immediate situation, producing motivational deficits (Evans and Stecker, 2004). According to Self-Determination Theory (Ryan and Deci, 2000), motivation and the degree of internalization of the behavior are keys to greater behavioral effectiveness, volitional persistence, and enhanced subjective well-being. Future research should consider these factors in order to deepen the knowledge about the effects of green office buildings on employees' outcomes.

Furthermore, we can see that most of the studies included in this review, especially in the area of sustainable office solutions aimed at reducing thermal and lighting costs (e.g., Kang et al., 2013; Konis, 2013; Zeiler et al., 2014; Pasut et al., 2015), focused only on measuring employees' well-being, and they did not complement these measures with evaluations of employees' performance. We suggest that this gap should be addressed in future research. Moreover, when assessing employees' performance, the measures should include both objective tests (e.g., computer based) and subjective assessments that consider different informants (e.g., the employees, their supervisor, or the clients) in order to avoid employees' leniency or self-deception in self-ratings, which has been shown to be especially pronounced in the case of general or trait judgments of performance (Heidemeier and Moser, 2009). These biases might occur because they address not only past behavior, but also respondents' expectations of current and future behavior (Wilson and Ross, 2001). Moreover, future studies should use a better conceptualization of performance and encompass its different facets, such as in-role performance, extra-role performance (e.g., citizenship behavior), and innovation or creativity.

Furthermore, we can see in the case of the studies that focus on the outcomes of green buildings that the research design plays an important role in the results. Specifically, longitudinal studies report the greatest proportion of significant positive relationships between sustainable solutions and well-being and performance, compared to cross-sectional and exploratory studies. Because longitudinal designs can be considered superior to the other two, and the number of longitudinal studies is still rather limited, we suggest that future research should focus on analyzing the impact of energy-efficient solutions using longitudinal designs. It is necessary for researchers using longitudinal designs to employ adequate and more robust analyses that allow them to make the most of these designs and model change, for example, with the use of repeated-measures ANOVA or Longitudinal Growth Curve Modeling.

In this context, we suggest that the study of the dynamics of the relationship between sustainable office solutions and employees' well-being and performance is an important avenue for research that has not been explored yet. The role of time and the dynamic relationships that fluctuate over time have been recognized as key elements in theory development (Pitariu and Ployhart, 2010) and essential for both well-being (see Sonnentag, 2015 for a review) and performance (Roe, 2014) at work, due to their dynamic nature. Indeed, these states are experiences that can fluctuate over time within the same person in response to the varying characteristics of the environment (Cervone, 2005; Ilies et al., 2007), due to their sensitivity to external stimuli (Xanthopoulou et al., 2012). The research in the area of the impact of sustainable office solutions on employees' well-being and performance should use relevant methods, such as experience sampling methods, in order to “*gain more insight into the temporal order of the underlying processes”* (Sonnentag, 2015) and capture the dynamics of the phenomena as they occur, instead of merely assuming that well-being and performance are global experiences (Xanthopoulou et al., 2009).

Finally, among the revised papers, we found that some authors considered other relevant criteria or outcomes of energy efficient solutions, in addition to well-being and performance, such as physiological outcomes (e.g., sleep quality, skin temperature; Newsham et al., 2013; Zeiler et al., 2014). In this review, we have focused on performance and well-being for the previously mentioned reasons: (1) interest for both organizations and the individual; (2) the volume of research on these outcomes in the area of decoupling in organizational context; and (3) the motivational value for employees and organizations to engage in actions to promote environmental sustainability, while, at the same time, maintaining well-being and performance at adequate levels. However, future studies should take into account other relevant outcomes and carry out a systematic review of the available evidence, as well as its theoretical and practical relevance.

The research results can serve as a useful reference for different stakeholders interested in implementing green building solutions, adopting energy-saving measures in offices, and improving employees' functioning. They especially suggest that organizations willing to implement energy-efficient solutions can do so on different scales: they can implement them locally at the employee level (e.g., user-in-the loop solutions), apply sustainable solutions to several work stations at the office level (e.g., lighting sources that produce more illuminance with less energy), or design or refurbish whole office buildings according to low-energy certification standards (e.g., LEED, Green Star, Energy Star). Thus, the results show that sustainable solutions are not only within the reach of large organizations and owners of office buildings who have the capacity to implement expensive global energy-efficient systems (e.g., HVAC). Indeed, this review indicates that there are also efficient local solutions (e.g., heated chairs) that can be implemented in companies that have a more limited budget and are renting office spaces, which can be an attractive option for an increasing number of start-up ventures starting with limited capital. The use of energy-efficient solutions is clearly an appealing way for businesses to improve their outcomes (e.g., employees' performance and well-being) while shrinking their energy-related costs and their ecological footprints. In addition, the results are relevant for designers and developers of energy-efficient technological solutions, suggesting that they should consider including features that offer some degree of manipulation in order to increase the perception of control by office users. Finally, the Socio-Technical approach (Rice, 1958) posits that social, technical, and environmental subsystems are interdependent organizational subsystems that should be optimally integrated to ensure maximum productivity and well-being (Baek et al., 2015). This means that companies aiming to modify the environment, instead of merely imposing new technologies, should take into account the employees, the machine-human fit, and the complexity of psychosocial phenomena at work, in order to ensure well-being and performance at work. This could be done through the active involvement of the employees in the process of organizational change.

The present review has some clear contributions because it further links innovative and sustainable building systems and technologies with improved IEQ by taking into account the human factor at work and adopting a user-centered approach that considers office users' well-being and productivity for the economic benefits of the organization. Specifically, a contribution of this review is that it focuses on research studies that used data provided by the participants, such as perception of comfort, instead of studies that used simulations or mathematical models without collecting any data from occupants. In this systematic review, we analyzed and summarized the results obtained in 34 empirical studies carried out in office buildings. In general, these studies provide some supportive empirical evidence for decoupling comfort and performance from an increase in energy consumption. Our results also provide evidence for the rationale of investing in energy-saving and sustainable solutions in office buildings. Finally, the presence of mediator variables (e.g., perceived control) suggests that occupant ratings of comfort and simulation-based estimations of comfort can have different dynamics, depending on these mediator variables.

## Conclusions

In general, the results of this systematic review suggest that the implementation of sustainable technologies and systems aimed at reducing thermal and lighting costs may not harm employees' well-being and performance, or it may even improve them. This, in turn, can result in increased productivity and, therefore, economic gains for businesses. Contrary to what might be expected, the results of this review suggest that human comfort is not directly linked to increased energy demands. In fact, achieving comfort conditions is not solely a matter of increasing lighting levels or rising/lowering temperatures in response to the uncomfortable temperature in the environment. Instead, it is a question of finding the optimal conditions and identifying psychological antecedents of sustainable behaviors. Most of the energy labels (MINERGIE, Passivehouse, LEED, BREEAM, DGNB) claim that energy efficiency goes hand in hand with higher comfort and better health, but systematic evidence supporting these claims was limited. This review has extended the knowledge about empirical evidence in order to encourage investors to consider these standards and take respective measures, such as energy-efficient air conditioning systems.

The evidence gathered in this review is relevant for the decoupling issue. The findings show that it is possible to maintain the well-being and productivity of office users while ensuring energy efficiency of office buildings. In this way, it is possible to decouple energy costs from well-being and productivity. Using energy-efficient solutions is a way for companies to improve their outcomes (e.g., employees' performance and well-being) while shrinking their ecological footprints (e.g., using less energy for lighting). For this reason, by decoupling human comfort from high energy consumption, we can achieve comfortable office spaces that are energy efficient (less CO_2_ emissions) and ensure healthy and productive work.

## Author Contributions

JMP, CM-C, LPM, and MWK contributed to the conception of the manuscript. MWK, LPM, DMV, and AS contributed to carrying out bibliographic search and analyses. MWK, LPM, and DMV contributed to the interpretation of data. MWK wrote the first draft of the manuscript. LPM, DMV, and JMP wrote sections of the manuscript. All authors contributed to manuscript revision, read, and approved the submitted version.

### Conflict of Interest Statement

The authors declare that the research was conducted in the absence of any commercial or financial relationships that could be construed as a potential conflict of interest.

## Footnotes

^1^European Parliament and Council Directive (2012). *On energy Efficiency, Amending Directives 2009/125/EC and 2010/30/EU and Repealing Directives 2004/8/EC and 2006/32/EC*. Available online at: http://data.europa.eu/eli/dir/2012/27/oj

## Notes

Annex 1 Search phrase used in the systematic literature search ((“decoupling” OR “energy saving” OR “energy consumption” OR “energy conservation” OR “energy efficien^*^” OR “low energy” OR “low-energy”) AND (“well-being” OR “stress” OR “physical complaints” OR “job satisfaction” OR “emotion^*^” OR “mood” OR “affect” OR “comfort” OR “absenteeism” OR “presenteeism” OR “turnover” OR “retention” OR “productivity” OR “performance” OR “engagement” OR “counterproductive”) AND (“employee” OR “personnel” OR “staff” OR “white collar”) AND (“research” OR “results” OR “participants” OR “subjects” OR “correlation^*^” OR “statistic^*^”)) AND AB office.
