# Supplementary material for: Decoupling Office Energy Efficiency From Employees' Well-Being and Performance: A Systematic Review
Source: Front Psychol. 2019 Feb 20;10:293. doi: 10.3389/fpsyg.2019.00293 (PMC6391329; doi:10.3389/fpsyg.2019.00293)
Supplement: Supplementary file 1 [file Table_1.pdf]

Appendix 1. Complete list of databases used in the literature search

Abstracts in Social Gerontology
Academic Search Complete
Academic Search Premier
Africa-Wide Information
Agricola
AHFS Consumer Medication Information
Alt HealthWatch
Alternative Press Index
America: History & Life
Applied Science & Technology Full Text (H.W. Wilson)
Art & Architecture Complete
Art Full Text (H.W. Wilson)
Art Index Retrospective (H.W. Wilson)
Associates Programs Source
ATLA Religion Database
Avery Index to Architectural Periodicals
Bibliography of Native North Americans
Biography Reference Bank (H.W. Wilson)
Biological & Agricultural Index Plus (H.W. Wilson)
Business Abstracts with Full Text (H.W. Wilson)
Business Source Complete
Business Source Premier
Caribbean Search
Central & Eastern European Academic Source
CINAHL Plus with Full Text
Communication & Mass Media Complete
Communication Abstracts
Computer Source
Computers & Applied Sciences Complete
Consumer Health Complete - EBSCOhost
Criminal Justice Abstracts with Full Text
eBook Collection (EBSCOhost)
EconLit
Education Full Text (H.W. Wilson)
Education Index Retrospective: 1929-1983 (H.W. Wilson)
Education Research Complete
Energy & Power Source
Entrepreneurial Studies Source
Environment Complete
ERIC
Essay and General Literature Index (H.W. Wilson)
European Views of the Americas: 1493 to 1750
Film & Television Literature Index with Full Text
Food Science Source
FuenteAcadémica
Funk &Wagnalls New World Encyclopedia
Garden
Landscape & Horticulture Index
Gender Studies Database
General Science Full Text (H.W. Wilson)
GreenFILE
Health and Psychosocial Instruments
Health Source - Consumer Edition
Health Source: Nursing/Academic Edition
Historical Abstracts
Hospitality & Tourism Complete
Human Resources Abstracts
Humanities Abstracts (H.W. Wilson)
Humanities Full Text (H.W. Wilson)
Humanities International Complete
International Bibliography of Theatre & Dance with Full Text
International Political Science Abstracts
International Security & Counter Terrorism Reference Center
Jewish Studies Source
Left Index
Legal Collection
LGBT Life with Full Text
Library
Information Science & Technology Abstracts with Full Text
Literary Reference Center
MAS Ultra - School Edition
MasterFILE Premier
MedicLatina
MEDLINE
MEDLINE with Full Text
Mental Measurements Yearbook with Tests in Print
Middle Eastern & Central Asian Studies
Middle Search Plus
MLA Directory of Periodicals
MLA International Bibliography
Music Index
National Criminal Justice Reference Service Abstracts
Newswires
Newspaper Source
Peace Research Abstracts
Philosopher's Index
Play Index (H.W. Wilson)
Political Science Complete
Primary Search
Professional Development Collection
PsycCRITIQUES
Psychology and Behavioral Sciences Collection
PsycINFO
Public Administration Abstracts
Public Affairs Index
Race Relations Abstracts
Readers' Guide Retrospective: 1890-1982 (H.W. Wilson)
Regional Business News
Religion and Philosophy Collection
RILM Abstracts of Music Literature (1967 to Present only)
Risk Management Reference Center
Science Reference Center
Shock & Vibration Digest
Short Story Index (H.W. Wilson)
Social Sciences Abstracts (H.W. Wilson)
SocINDEX with Full Text
Sociological Collection
SPORTDiscus with Full Text
Teacher Reference Center
The Serials Directory
TOPICsearch
VenteetGestion
Vocational and Career Collection
Vocational Studies Premier
Waters & Oceans Worldwide
Women's Studies International
L'Annéephilologique
